# Supplementary material for: Are carbon emissions trading and green financial instruments synergistic? -Comprehensive quantitative research based on content analysis
Source: PLoS One. 2024 Mar 7;19(3):e0298601. doi: 10.1371/journal.pone.0298601 (PMC10919601; doi:10.1371/journal.pone.0298601)
Supplement: S1 Data — (ZIP) [file pone.0298601.s001.zip › Data/地级市Z_F工作报告.zip!/╔╜╢1⁄2/╟α╡║╩╨/└┤╫╘╣1⁄2╓┌║┼í╛┬φ┐╦╩2╛▌═°í┐_2004.docx]

各位代表:

　　现在，我代表市人民政府向大会做工作报告，请予审议，并请各位政协委员和其他列席会议人员提出意见。

　　一、2003年政府工作的回顾

　　2003年是本届政府施政的第一年。在中共青岛市委的正确领导下，在市人大、市政协的监督和支持下，在历届市领导班子打下的良好基础上，我们紧紧依靠全市人民，坚持以邓小平理论和“三个代表”重要思想为指导，全面贯彻落实党的十六大精神和省委、省政府的各项战略部署，按照中共青岛市第九次代表大会提出的目标任务，解放思想，开拓进取，扎实工作，较好地完成了市十三届人大一次会议确定的各项任务，实现了国民经济的快速发展和社会事业的全面进步。

　　（一）构建可持续发展的经济体系取得新进展，经济增长质量不断提高。初步核算，全年实现生产总值1780亿元，增长15.9％，是1995年以来的最高增幅；完成地方财政一般预算收入120亿元，增长23.9％。

　　四大产业基地建设成效显著。大炼油、汽车等项目前期工作取得突破，海西湾造修船基地列入国家规划，浦项冷轧不锈钢板项目主体工程完工，前湾港三期工程部分通过国家验收。港口吞吐量达到1.4亿吨，集装箱运量424万标箱。海洋、信息、软件等产业加速成长，高新技术产业产值首次突破1000亿元，占限额以上工业总产值的比重达到39.7％。

　　城郊经济对全市发展的拉动作用增强。五市实现生产总值占全市的比重达到49.3％。农业结构进一步调整，粮经比例由上年的56.5：43.5调整为53.1：46.9。农村城镇化水平提高1.5个百分点。农村税费改革稳步推进，农民减负率达到42.4％。

　　服务业发展呈现新态势。克服非典的影响，旅游经济迅速回升，旅游市场促销收效明显，崂山巨峰、九水十八潭、海底世界等旅游项目投入使用。物流业快速崛起，伊藤忠、沃尔玛、百安居、欧倍德、伊都锦等世界著名企业相继来青投资。金融证券保险业稳定发展，引进韩国中小企业银行等15家金融和保险中介机构。

　　（二）构筑特色鲜明的现代化国际大城市框架迈出新步伐，奥运筹备工作顺利展开。全面实施“三点布局、一线展开、组团发展”的战略构想，编制了城市发展概念规划和近期建设规划，市内四区的控制性规划覆盖率达到98％。同三线高速公路青岛段、前湾港疏港高速公路、东西快速路二期、滨海步行道市区段和第一、第三海水浴场改造等工程竣工。流亭机场扩建一期工程即将交付使用。滨海公路实验段开工建设。中山路商贸区综合改造完成局部拆迁。

　　城市环境综合整治取得成效。团岛流域截污工程进展较快，海泊河、团岛污水处理厂等中水利用示范项目完成。城市空气质量继续改善。建成区绿化覆盖率达到37.5％。获得“中国人居环境奖”。公用事业市场化进程加快，麦岛污水处理厂扩建等项目正式签约。城市管理综合执法体系不断完善。

　　青岛奥帆委正式成立。北海船厂搬迁工作完成计划进度。组织开展了奥运文化节。编制完成了《奥运行动规划》和《迎奥运亮化总体方案》。《奥运会青岛国际帆船中心赛时规划》已经北京奥组委和国际帆联批准。

　　（三）提升城市核心竞争力取得新成效，发展环境继续优化。政府“五项工程”和“四型机关”建设深入推进，机关作风整顿活动持续开展。修订了《青岛市人民政府工作规则》，出台了《重大社会公共事项决策听证办法》和《行政过错责任追究暂行规定》，建立了政府部门规范性文件事前备案审查和行政执法社会监督员制度。邀请人大代表、政协委员列席市政府常务会议。完成了第二轮行政审批事项的清理调整，向社会公布了所有行政性审批和收费事项。“我为青岛发展献计策”市民月活动收到良好效果。办理人大代表议案、建议和政协委员提案1490件。

　　城市创新体系建设逐步加强。全年取得重要科技成果544项，授权专利1500项。国家海洋科研中心等一批重要科研基地的建设加快。被国家列为技术标准试点城市。实施科技专项计划效果明显。各级各类教育健康发展，高等教育、职业教育、民办教育办学规模继续扩大，教学质量稳步提高。全面实施“111人才引进”工程，引进各类人才1.93万名。引进外国专家132人次，国外智力项目97项。

　　（四）对外开放工作开创新局面，各项改革不断深化。全年外贸出口103亿美元，实际利用外资40亿美元，提前两年完成“十五”计划目标。外商投资环境改善，口岸通关速度加快。引进投资1亿美元以上项目6个，世界500强企业新投资项目17个。引进韩国、日本投资比上年实现翻番。建成跨国采购促进中心，青岛出口加工区通过国家验收。出口结构进一步优化，高新技术产品和机电产品出口增长较快。广泛开展与国外城市政府和民间的交流交往，港澳台侨工作取得积极成果。加强国内经贸合作，引进国内各类投资增长47％。

　　年初确定改制的110户国有企业基本完成。国有大企业股份制改造和战略重组取得实质性进展，中小企业改制退出全面展开。为减轻企业负担，降低了国有集体企业养老保险统筹的比例。民营经济发展加快，全年实现增加值521亿元，增长23％。50户民营企业年营业（销售）收入过亿元。规范了行政机构编制管理，全市议事协调和临时机构精简74％。启动了事业单位人事制度改革。

　　（五）精神文明建设扎实推进，各项社会事业健康发展。以“培育城市精神，提高文明素质”为主题开展文明创建活动，以“诚信、博大、和谐、卓越”为核心理念的城市精神正在成为广大市民的共识。文化事业持续繁荣，6部作品荣获“五个一工程奖”。体育工作再创佳绩，在第五届城运会上金牌和奖牌总数创历届最好水平。广电事业稳步发展，数字电视的建设走在全国前列。气象和防震抗灾工作得到加强。新闻、出版、民族、宗教、档案、史志等工作取得新进展。国防教育、民兵预备役、人防工作取得新成绩，连续五次被命名为“全国双拥模范城”。

　　强化社会治安综合治理，为期两年的严打整治斗争收效显著。人民群众对社会治安的满意率达到95％，比上年提高6.6个百分点。同国内外敌对势力和“法轮功”等邪教组织的斗争取得新成果。信访工作秩序良好，化解社会矛盾成效明显。深入开展全民普法教育，社会法治环境有所改善。

　　（六）人民生活水平进一步改善，就业和社会保障工作力度加大。城市居民人均可支配收入达到10075元，农民人均纯收入达到4530元，分别增长15.5％和8％。提高了城乡居民最低生活保障标准。基本解决了国有集体企业拖欠职工工资和建设领域当年拖欠农民工工资的问题。经济适用房竣工60.8万平方米。市区新增供热面积150万平方米，新增天然气用户1.5万户。市政府年初确定的12件实事，除有的项目因征地拆迁和选址论证问题暂缓实施外，已完成计划目标。全年实现就业11.2万人，城镇登记失业率为3.75％。下岗职工出中心工作全部完成。政府社会保障支出9.3亿元，增长13.6％。企业养老保险覆盖面进一步扩大，建立农村养老保险制度试点工作取得成效。以大病统筹为主的新型农村合作医疗制度覆盖200万人。加强了食品安全保障措施，努力营造群众放心、安全、健康消费的环境。民政、老龄、残疾人、妇女、儿童工作健康发展。

　　去年春天，面对传播迅速、危害严重的非典疫情，在市委的坚强领导下，按照“四个全力做到”的工作要求，落实“堵、防、教、治”的防控措施，万众一心，众志成城，取得了防治非典的阶段性重大胜利。同时，制订出台了《突发公共卫生事件应急反应长效机制建设方案》、《突发公共卫生事件应急办法》，公共卫生体系建设得到加强。

　　各位代表！过去的一年，是不平凡的一年。一年来青岛经济和社会发展取得的成就，充分展现了全市人民解放思想、干事创业、求真务实、加快发展的进取精神；充分展现了全市人民在危难和挑战面前团结拼搏、勇往直前、敢于胜利的可贵品质；充分展现了我们这个城市诚信和谐、追求卓越、奋发向上的时代风采。这些成就的取得，是党中央、国务院、省委、省政府和市委正确领导的结果，是市人大、市政协监督和支持的结果，是全市人民不懈努力的结果，是社会各界通力合作、广泛参与的结果。在此，我代表青岛市人民政府，向为青岛发展做出无私奉献、付出辛勤劳动的全市人民，表示崇高的敬意！向给予政府工作大力支持的人大代表和政协委员，向各民主党派、工商联、无党派人士和社会团体，向人民解放军驻青部队和武警官兵，向中央和省驻青单位，向所有关心、支持青岛发展的海内外朋友们，表示诚挚的感谢！

　　回顾一年的工作，我们清醒地看到，在前进中还面临着许多亟待解决的问题。主要表现在：经济发展的结构性矛盾突出，产业集群和服务业发展不快；土地资源的有效利用不够，城市规划、建设和管理还不适应发展的要求；人才总量不足，结构不合理，城市创新能力还不强；社会保障和就业压力较大，由于农民土地征用、城市居民拆迁、企业改制破产等带来的社会矛盾的化解机制有待完善；解决“三农”问题任务艰巨，部分群众生活比较困难；城市中低收入家庭住房条件有待改善，公共服务设施仍较薄弱；一些政府工作人员法律素质不高、全局观念淡漠、服务意识不强，形式主义、官僚主义严重，少数人行政不公、为政不廉，甚至以权谋私、贪污腐败。这些问题我们将高度重视，采取切实措施加以解决。特别是对关系群众切身利益的问题，要集中力量认真解决好。

　　二、2004年政府工作的总体要求和主要任务

　　今年政府工作的总体要求是：以邓小平理论和“三个代表”重要思想为指导，深入贯彻党的十六大和十六届三中全会精神，全面落实省委、省政府各项战略部署和中共青岛市第九次代表大会提出的目标任务，坚持科学的发展观和正确的政绩观，坚持求真务实、真抓实干，全力推进“三件大事”，突出结构调整、改革开放、科技创新三个关键，高度重视解决“三农”问题和关系人民群众切身利益的问题，大力加强政治文明和精神文明建设，深化依法行政和施政创新，努力实现经济和社会的全面、协调、可持续发展。

　　今年国民经济和社会发展主要预期指标是：全市生产总值增长13％；地方财政一般预算收入增长14％；城镇登记失业率控制在4.3％以内；城市居民人均可支配收入增长10％，农民人均纯收入增长7％。重点做好以下十个方面工作：

　　（一）切实加快产业集群和制造业基地建设

　　抓好15个投资过10亿元重大项目，争取大炼油、一汽乘用车、海西湾造修船、电厂扩建等项目开工建设。结合落实省建设胶东半岛制造业基地战略部署，制定产业基地布局及产业链发展规划，加快建设以海尔、海信、澳柯玛、朗讯、浪潮等为核心的电子家电产业群；以炼油、芳烃等为核心的石化产业群；以轿车、卡车、客车、专用车为核心的汽车产业群；以集装箱船、特种船、游船游艇为核心的造修船产业群；以港口运输、集装箱作业、现代物流为核心的港口产业群。

　　发挥大企业在产业集聚、招商引资、技术创新等方面的平台作用，落实和完善各项支持政策。重视发展中小企业，构筑专业化分工、系统化生产的协作体系。继续实施名牌战略，引导生产要素向优势企业集聚。坚持走新型工业化道路，加快利用高新技术改造传统产业，鼓励开发有自主知识产权的核心技术，推动行业技术升级。实施好20个信息技术推广应用项目，加强企业信息化建设。

　　（二）高度重视解决“三农”问题

　　按照统筹城乡发展的要求，坚持“多予、少取、放活”的方针，坚持城乡统筹发展，进一步调整农业结构，深化农村改革，加大农业投入，强化对农业的支持和保护，推进农村城镇化、工业化和农业产业化，做好农民的社会保障、减轻负担和农村劳动力的教育培训、有序转移工作，努力增加农民收入。

　　搞好城乡统筹规划和产业统筹布局，加速农村城镇化和工业化。编制完成《青岛市滨海公路沿线城市组团总体规划》，并严格控制好沿线两侧土地规划使用，开工建设滨海公路北段工程。年内五市驻地、重点镇的详规覆盖率分别达到90％和70％以上。重视农村经济和社会的协调发展，整合现有各项支农投资，提高资金使用效率，增加水利、道路、生态等基础设施的投入。实施五镇二岛集中供水工程。促进大工业辐射带动，工业基地和产业集群的规划要统盘考虑五市，采取区域分工、错位发展、构筑产业链等措施，增强加工制造业配套能力。促进城乡服务业的协调发展，优化区市驻地和重点镇、沿海小城镇服务业布局。

　　继续推进农业结构调整和产业化进程。制定实施优势农产品区域布局规划，扩大优质专用农作物生产。培植一批高水平的创汇农业、都市农业、观光农业和特色农业示范区。保持适度的粮食综合生产能力，壮大水产、畜牧、蔬菜、花卉、林果、粮油六大产业。通过外向型农业拉动、城市大工业参与、九联模式推广等措施，提高农业产业化经营水平。各级财政要安排支持农业产业化发展的专项资金，采取贴息贷款和以奖代补等形式，扶持农产品加工龙头企业做大做强。加强对新办中小型农副产品加工企业的创业扶持和服务。安排农业发展基金，重点扶持高水平农业生产基地和农业园区建设。完善农业的检验检测、安全检测及质量认证体系，加快无公害农产品、绿色食品、有机食品的产业化。

　　认真落实中共中央、国务院《关于促进农民增加收入若干政策的意见》，加大对农业的政策支持和资金投入。完善农村税费改革，降低农业税税率，有条件的地方年内取消农业税征收。把对粮食流通环节的间接补贴改为对农民的直接补贴。加强农村土地承包管理，稳定和完善土地承包关系。全面实行涉农税收、价格、收费公示制度。依法治理“三乱”现象，切实减轻农民负担。重视解决好农村剩余劳动力的转移问题，统筹规划城乡劳动力资源，有序吸收农民工进城务工经商。取消农民进城就业的歧视性规定和不合理收费，继续清理拖欠农民工工资等问题，依法保护进城务工农民的合法权益。从今年起，义务教育阶段的农村进城务工人员子女享受“市民待遇”。

　　（三）突出抓好招商引资和对外贸易

　　积极扩大利用外资规模，不断提高引资质量和水平。完善招商机制，创新招商方式，规范招商秩序，确保完成全年目标。利用优势产业、大企业和国家、省级园区，实现产业集群招商的新突破。重点抓好对韩国、日本、德国、台湾等国家和地区的招商，力争实现大幅增长。加大对世界500强的招商力度。鼓励现有外资企业增资扩股。抓好协议项目和合同项目的跟踪落实，提高资金到位率和开工投产率。引导外资通过收购兼并、参股控股等方式，参与国有集体企业的改组改造。积极引进物流、金融、保险、商贸、旅游、中介咨询等服务业项目。继续扩大国内招商引资，采取多种鼓励措施，吸引更多的国有大企业和民营企业来青岛发展。

　　努力调整出口商品结构，积极开拓国际市场。扩大加工贸易规模，引导加工贸易转型升级。用足用好出口加工区政策，吸引更多的加工企业到园区投资发展。提高国家和省级园区的出口比重，提高机电产品和高新技术产品的出口比重。支持民营企业增加出口。做好跨国采购促进工作。组织好企业的反倾销应诉，有效应对国际贸易摩擦。统筹安排财政资金，确保地方出口退税及时足额支付。鼓励有条件的企业走出去，推动境外投资与合作。

　　进一步优化开放环境，重点解决好商务成本高、配套能力弱等问题。建成口岸电子商务信息平台，办好外商投资行政服务大厅，完善“一门式、一条龙”服务体系。加快“大通关”建设，提高口岸验放速度。争取国家批准“区港联动”试点。加强外事、侨务、对台工作。完善外商投诉的及时处置、责任追究、案例通报等制度。继续清理规范外商投资的审批事项和审批环节。切实改善外国人居住、医疗、子女入学等条件。

　　（四）全面推进各项改革和民营经济发展

　　深化国有资产管理体制改革和国有企业改革。利用两年时间，基本完成国有资本结构调整的主要任务。研究建立国有资本经营核算制度，加强国有资本收益和转让变现收入的管理。做好国有资产保值增值考核奖惩工作。推进国有企业股份制改造，推动国有持股、控股企业国有股的减持或退出，加快中小型企业的国有资本退出。完善企业法人治理结构，鼓励企业转换内部经营机制。整合产权市场，建立流转顺畅的产权交易机制。分类改革重组资产经营公司，组建综合性国有资本营运机构。强化审计监督，做好预算执行审计，严格固定资产投资项目的审计，全面实行党政机关和国有企业领导人员的经济责任审计。

　　深化政府投融资体制改革。规范政府投资行为，经营性项目向社会资本和海外资本开放。深化国有投资公司的股份制改造，鼓励其参与重大基础设施和公益性项目的投融资。强化对政府投资的监督管理。深化农村信用社改革，力争在省内率先成立市农村合作银行。实施新一轮行政管理体制改革和政府机构改革。积极稳妥地推进事业单位分类改革和管理。抓好政府、企业和个人信用体系建设。

　　放手发展民营经济。全面落实促进民营经济发展的各项政策，清理、废除影响和限制民营经济发展的各种规定，进一步降低门槛、放宽准入、完善服务、优化环境。支持非公有资本进入法律法规未禁入的基础设施、公用事业等行业和领域。实施民营企业“百强计划”，支持一批企业在优势产业和领域做大做强。继续实施“小老板创业”工程，鼓励归国留学人员、科技人员和高校毕业生等创办高新技术企业。完善扩大民间投资的配套措施，鼓励多渠道筹集担保资金、多形式建立担保机构，为民营企业发展提供金融支持。

　　（五）提升服务业发展水平

　　制定全市服务业发展规划。以前湾港国际物流园和流亭空港物流区为重点，培育一批新的物流项目。打造会展经济品牌，办好APEC中小企业技术交流展览会、电子家电博览会、啤酒节、海洋节、时装周等大型展会，争取新的国家级大型展会定点承办。抓好国际会展中心二期工程建设。

　　以建设区域性金融中心为目标，积极发展金融保险业。拓宽金融业务领域，完善金融服务体系。加快企业境内外上市，进一步规范已上市公司行为。争取国家保监会在青设立监管机构。大力引进内外资银行、外资保险机构和金融保险中介机构。继续推进金融安全区创建活动，逐步扩大试点范围，努力防范化解金融风险。

　　培育消费热点，推动消费结构升级。完善城乡商业网点网络和商品市场体系。以建设国际海滨度假城市和争创中国最佳旅游城市为目标，实施海军博物馆、极地海洋世界、石老人浴场的改造，连通和延伸滨海步行道，推进薛家岛、琅琊台等度假区风景区的建设。加大国际旅游促销，整顿旅游市场秩序。启动“社区服务中心信息系统”工程，促进城市商贸流通业向农村延伸，完善社区商业服务体系。

　　（六）坚持科教兴市和可持续发展

　　加快科技创新体系建设。完善以高校科研院所及重点实验室为龙头、企业为主体的塔型研发体系，推进源头创新、工艺创新和技术集成创新。做好国家海洋科学研究中心组建工作。集成技术资源、设备资源和人才资源，建设具有核心竞争力的创新群体，领衔和承接国家重大战略性科研项目。促进企业自主创新，建设面向中小企业信息化建设的服务托管平台。实施技术标准战略，完善技术标准信息平台。加强科技支撑服务，发展风险投资业务，加快风险投资等中介机构建设。发挥国家级园区的示范带动作用，着力培育海洋、新材料和软件三大高新技术产业。

　　优化教育资源配置和布局。通过新建、扩建和办分校等方式，扩大优质教育资源。加强城区条件较好的学校对农村薄弱学校的帮扶，实现联手发展。逐步提高农村高中段教育规模，力争用2到3年时间使高中段入学率达到90％。职业教育与成人教育要进一步提高办学质量。支持高等学校发展，做好引进国内著名高校和科研院所的工作。

　　深入实施三项基本国策。强化人口与计划生育目标责任制考核与管理，建立人口出生预报制度，加强流动人口的计划生育管理与服务，开展“关爱女孩行动”。全年人口自然增长率控制在4.96‰以内。全面启动生态市建设，加强生态环境保护。发展循环经济，推广清洁生产，扩大绿色消费。突出抓好海域环境保护、重点流域污染防治、改善大气环境质量、医疗废物处理等工作。高度重视胶州湾的污染综合治理。实施电厂海水脱硫工程，继续淘汰10吨以下燃煤锅炉。加快后备水源建设，抓紧官路和沐官岛水库建设的前期工作。扩建麦岛污水处理厂，实施仙家寨老水厂二期改造。下大力气整治前海一线排污口，解决云霄路饮食一条街的污水排放问题，搞好团岛流域截污和中水回用工程及海水利用。进一步整顿规范土地市场秩序，严厉打击各种土地违法行为。加强土地利用总体规划管理，严把土地利用年度计划、建设项目用地预审和土地审批关。严格执行基本农田保护制度。努力保持耕地总量动态平衡和耕地占补平衡。依法实行统一征地，加强和改进土地征用工作。建立征地与储备结合的制度，严格执行经营性土地招标拍卖和挂牌出让制度。积极争取国家和省的支持，依法保障经济建设和社会发展用地。

　　加速构筑人才高地。树立科学的人才观，围绕“培养、吸引、用好、留住”等环节，切实抓好人才队伍建设。大力引进中高层次的经营型、创业型、技能型人才和国内外智力，完善技术、知识、管理等要素参与分配等制度。出台人才工作的有关政策，开通引进急需紧缺和特殊人才的“绿色通道”，用事业造就人才，用环境凝聚人才，用机制激励人才，用法制保障人才，实现区域人才一体化开发和有序流动，为经济社会发展提供人才保证和智力支持。

　　（七）积极做好城市建设和奥帆赛筹备工作

　　加快城市规划的编制和城市建设。着手修编《青岛市城市总体规划》。完成城市主干道管网综合规划和八大关综合整治规划。做好控制性详细规划编制工作，在市内七区要全部覆盖建设用地的范围。开工建设流亭机场扩建二期、胶济铁路电气化改造、李村河中下游和板桥坊河等河道综合整治，改造辽阳路、遵义路等重点路段，继续推进中山路商贸区改造。实施绿化工程，建设城市森林景观、绿色海岸景观和城区园林景观，改造市区主干道两侧绿化设施。实施对八大关景区、历史优秀建筑和文化名人故居的保护与改造。抓好东西快速路周边环境美化和综合整治。

　　切实加强城市管理和经营。按照“管理重心下移”和“责权统一”的原则，深化城市管理体制改革。完善城市管理综合执法体系，全面推行辖区执法巡查责任制。加大违法建筑拆除力度，解决环境脏乱差等问题。吸引各类投资主体参与市政公用设施的建设与经营。

　　全面实施奥运行动规划。完成北海船厂搬迁和奥帆赛基地必备项目的规划设计，年内确保开工建设。完成原有码头岸线改造及赛场周边水域清淤等工程。启动赤潮防治和水文、气象监测以及国家级监测的相关工作。广泛开展奥运宣传，继续办好奥林匹克文化艺术节等重大活动，营造全民“参与奥运、支持奥运”的浓厚氛围。

　　（八）加强精神文明建设和民主法制建设

　　以创建全国文明城市为目标，塑造以“诚信、博大、和谐、卓越”为核心理念的城市精神，实施“人文奥运”工程，开展“做文明市民、建文明城市、办成功奥运”活动。深化创建学习型城市工作，建立全民参与的社会化、开放式、广覆盖的学习体系，推动城市人文环境改善和整体文明程度明显提高。围绕创建现代文化名城，抓紧青岛大剧院建设工作，改造现有影剧院，完善各级图书馆、文化馆等公益文化设施，每个社区都建有综合性多功能的室内文化活动场所。实施精品战略，繁荣文艺创作。精心筹办“中国青岛国际小提琴比赛”。深化文化体制改革，制定出台文化产业政策，促进文化产业发展。重视社会科学研究，促进哲学社会科学发展。加快“数字青岛”建设，普及数字电视。深化国防教育，增强国防观念，积极推进民兵预备役和人防建设，加强军地双方的协调配合，深入开展双拥共建活动。

　　加强民主与法制建设。各级政府要自觉接受人大及其常委会的监督。重视和发挥人民政协、各民主党派及工商联在政治协商、民主监督和参政议政中的作用。做好人大代表和政协委员提出的各项议案、建议、提案办理工作的督查落实。充分发挥工会、共青团、妇联等社会团体的作用。深入开展普法教育，增强市民法律意识，提高市民法律素质。推进以民主选举、民主决策、民主管理、民主监督为主要内容的基层民主政治建设，推进政务、厂务、村务“三公开”。发展法律服务中介机构，完善法律服务体系。

　　全面建设“平安青岛”。全力维护政治安全、社会安全、信访安全和生产安全，广泛开展建设“平安区”、“平安市”、“平安乡”活动。进一步加强社会治安综合治理，坚持工作重心下移，强化基层基础工作，把“严打”整治斗争、治安防范、矛盾纠纷排查调处、治安管理、法律服务等工作，落实到城乡基层单位。严厉打击刑事犯罪活动，依法从重从严打击各类危害市场经济秩序的犯罪活动，严密防范和依法打击境内外敌对势力的破坏活动。深入开展同“法轮功”等邪教组织的斗争。认真排查调处各种社会矛盾，把人民调解、行政调解、司法调解结合起来，努力消除不稳定因素。高度重视做好人民群众来信来访工作。严格落实安全生产管理措施和责任制，坚决遏制重特大安全生产事故的发生。

　　（九）千方百计为人民群众办实事

　　坚持以人为本，努力提高人民群众的生活水平和生活质量，从群众最关心、最需要的问题入手，诚心诚意地办实事、做好事，着力解决关系人民群众切身利益的实际问题。

　　认真做好就业再就业工作。坚持劳动者自主择业、市场调节就业和政府促进就业的方针，全面落实税费减免、小额贷款、社保补贴、岗位补贴等促进就业政策。加强过渡性就业、政策性扶持岗位开发、社区就业岗位开发等工作，完善就业困难群体帮扶机制。高度重视国有企业改制后的职工安置工作。探讨建立城乡统筹就业体制试点，逐步形成城乡一体的就业管理和服务体系。全年实现就业9万人，新增政策扶持性就业岗位7500个，开发正规就业岗位6.5万个、灵活就业岗位3.5万个。整合社会各方面培训资源，加强失业人员、农村劳动力、在职职工的技能培训。年内综合劳动力市场竣工，高级技工学校争取开工。

　　继续完善社会保障体系。加大社会保险征缴、清欠、扩面工作力度，确保企业离退休人员养老金按时足额发放。进一步降低国有集体企业养老保险统筹比例。认真解决国有企业拖欠离退休人员四项补贴问题。完善医疗保险政策和管理，积极探索医疗保险与社区医疗服务相结合的途径。健全失业保险运行机制，抓好失业保险基金征缴。推进企业退休人员社会化管理服务，切实搞好城乡居民最低生活保障制度的落实。全面实施《城市困难群众医疗救助制度》，扩大农村养老保险制度试点范围，探索建立失地农民社会保障长效机制。

　　加快构建功能完备、资源共享、协调统一的城市公共安全应急体系。抓好公共卫生安全应急体系建设，增强突发性公共卫生事件、重大动物疫情的应急处置能力。提高公共卫生服务水平，大力开展社区卫生服务，全面建立以大病统筹为主的新型农村合作医疗制度，完善农村三级医疗预防保健体系，逐步解决特困农民“看病难”问题。积极构建全民健身服务体系，广泛开展以迎奥为主题的系列活动，推进“帆船之都”建设。实施“青岛奥运争光计划”，提高竞技体育发展水平。关心老年人的健康和生活，重视青少年成长成才，促进妇女、儿童事业和残疾人、慈善事业的全面发展。做好民族宗教工作。年内理顺各区、市的红十字会管理体制。

　　认真解决关系人民群众切身利益的问题。增加多层次住房供给，缓解市民“买房难”，建设经济适用房200万平方米，竣工60万平方米。改善和解决好居民用水、集中供热、管道燃气等方面存在的问题。进一步提高供热质量，市区新增供热面积100万平方米。有计划地对市区50年以上供水管道进行改造。实施大沽河下游综合治理二期工程。在重点区域、道路和景点规划建设停车场、过街通道和公厕。抓好“五纵四横”、“亮化大环线”和前海一线夜景亮化美化，对市内四区无路灯的市政道路全部安装路灯。开工建设市体育馆。扩建市老年大学。建立义务教育财政补助制度，解决城乡特困家庭子女“上学难”问题。深入实施“三绿工程”，严厉打击食品、药品、农业生产资料等领域的制假售假行为。

　　各位代表！人民政府是代表人民利益、为人民服务的政府。我们要始终把为人民群众办实事、办好事作为义不容辞的责任和义务。虽然目前我们的财力还不够雄厚，人民群众生活中遇到的困难和需要解决的问题还很多，有些问题短时间内难以完全解决。但是，只要我们坚持以人为本，按照统筹发展的要求，认真去对待、努力去解决，就能够得到广大人民群众的理解和支持。我们在工作中将集中力量，制定方案，调配资源，创造条件，尽力解决关系群众切身利益的实际问题，使广大人民群众在经济和社会发展中得到更多的利益和实惠！

　　（十）切实抓好政府自身建设

　　政府自身建设要体现立党为公、执政为民的要求，大力弘扬求真务实精神，大兴求真务实之风，坚持“为民、务实、清廉”，推进依法行政和施政创新，努力营造一流的政务环境。

　　加快职能转变，着力提高执政能力。政府职能转变要适应统筹发展的要求，更加注重经济调控、市场监管、社会管理和公共服务。政府的政策支持和财力投入的重点，进一步向社会事业、公共设施、扩大就业、完善社会保障体系、改善困难群众生活等方面倾斜。深化政府管理体系“五项工程”和“四型机关”建设，强化监督约束和工作考核。继续抓好行政审批制度、财政管理体制、干部人事制度等改革。切实提高政府工作人员特别是领导干部科学判断形势、驾驭市场经济、应对复杂局面、依法行政和总揽全局的能力。

　　全面推进依法行政。十届全国人大常委会第四次会议通过的《行政许可法》，对政府工作提出了新的更高的要求。政府系统要进一步组织好《行政许可法》的学习、宣传和培训。抓紧清理现行有关行政许可的政府规章和实施主体。7月1日起，凡是与《行政许可法》相抵触的行政许可规定，一律停止执行；凡是未经法定公布的机关和组织，不得实施行政许可。严格按照法定权限和法定程序行使权力、履行职责，积极推进行政流程再造和管理服务创新。继续完善行政监督，落实行政问责制、执法责任制、行政和执法过错责任追究制。切实加强作风建设。把求真务实、真抓实干作为转变作风的关键。加强对政府工作人员的思想教育，摆正同人民群众的关系，树立科学的发展观和正确的政绩观，不断提高求真务实的自觉性。抓好各项工作的落实。围绕中共青岛市委确定的目标任务，研究解决好经济社会发展中的重大问题、紧迫问题和突出问题。广泛开展访贫问苦、访贤问能、到基层谈心的“三访谈”活动。完善决策目标、执行责任、考核监督“三个体系”，把各项工作落到实处。建立和健全各项制度。坚持和完善学习调研、民主集中制、民主决策、政务公开等制度，坚持和完善重大事项社会公示听证和新闻发言人制度，使人民群众享有更多的知情权、参与权和监督权，进一步增强行政透明度。

　　深入推进反腐倡廉工作。按照“八个坚持、八个反对”和“两个务必”的要求，落实重大事项报告、民主评议制度和“一岗双责”廉政责任制。把纠正群众反映强烈的不正之风作为廉政建设的重要内容，抓紧抓实。强化对权力行使的制约和资金使用的监控，严格执行建设工程招投标、经营性土地使用权出让“招、拍、挂”、产权交易“公开、公平、公正”、政府采购招标等四项制度。坚决禁止搞劳民伤财的“政绩工程”和“形象工程”，坚决反对弄虚作假和浮夸虚报。完善预警、重点检查和定期检查机制，建立健全教育、制度、监督并重的惩治和预防腐败的工作体系。

　　各位代表！当前，青岛的发展机遇与挑战并存。完成既定目标，实现全面、协调、可持续发展，任务艰巨，责任重大。让我们更加紧密地团结在以胡锦涛同志为总书记的党中央周围，高举邓小平理论伟大旗帜，全面贯彻“三个代表”重要思想，在中共青岛市委的坚强领导下，解放思想，干事创业，求真务实，真抓实干，把青岛的改革开放和现代化建设事业不断推向前进！
